# HTLV-1 Tax Mediated Downregulation of miRNAs Associated with Chromatin Remodeling Factors in T Cells with Stably Integrated Viral Promoter

**DOI:** 10.1371/journal.pone.0034490

**Published:** 2012-04-04

**Authors:** Saifur Rahman, Kevin Quann, Devanshi Pandya, Shruti Singh, Zafar K. Khan, Pooja Jain

**Affiliations:** Department of Microbiology and Immunology, Drexel Institute for Biotechnology and Virology Research, College of Medicine, Drexel University, Philadelphia, Pennsylvania, United States of America; George Mason University, United States of America

## Abstract

RNA interference (RNAi) is a natural cellular mechanism to silence gene expression and is predominantly mediated by microRNAs (miRNAs) that target messenger RNA. Viruses can manipulate the cellular processes necessary for their replication by targeting the host RNAi machinery. This study explores the effect of human T-cell leukemia virus type 1 (HTLV-1) transactivating protein Tax on the RNAi pathway in the context of a chromosomally integrated viral long terminal repeat (LTR) using a CD4^+^ T-cell line, Jurkat. Transcription factor profiling of the HTLV-1 LTR stably integrated T-cell clone transfected with Tax demonstrates increased activation of substrates and factors associated with chromatin remodeling complexes. Using a miRNA microarray and bioinformatics experimental approach, Tax was also shown to downregulate the expression of miRNAs associated with the translational regulation of factors required for chromatin remodeling. These observations were validated with selected miRNAs and an HTLV-1 infected T cells line, MT-2. miR-149 and miR-873 were found to be capable of directly targeting p300 and p/CAF, chromatin remodeling factors known to play critical role in HTLV-1 pathogenesis. Overall, these results are first in line establishing HTLV-1/Tax-miRNA-chromatin concept and open new avenues toward understanding retroviral latency and/or replication in a given cell type.

## Introduction

Human T-cell leukemia virus type 1 (HTLV-1) was the first human retrovirus isolated from a patient with T-cell lymphoma [1], [2] and is known to cause two major diseases: a progressive lymphoma designated adult T-cell leukemia (ATL) [3], [4] and a neuroinflammatory disease called HTLV-1-associated myelopathy, also referred to as tropical spastic paraparesis [5], [6]. Although about 10 million to 20 million people are infected with HTLV-1 worldwide [7], only 5% of such infected individuals develop ATL or tropical spastic paraparesis, whereas 95% remain asymptomatic carriers [8], [9], [10]. It is still not fully understood why only a small percentage of the infected individuals develop the disease [11]. The virus preferentially targets CD4^+^ T cells [12], but other secondary cell types such as CD8^+^ T cells [13], cells of the monocyte-macrophage lineage, and dendritic cells [14], [15] as well as those belonging to the resident CNS cell population [16] are also known to be infected. Once the virus genome has been introduced into the target cell, it is reverse transcribed and integrated semi-randomly into the host genome as a provirus [17]. The provirus, like eukaryotic DNA, is coiled with histone and non-histone proteins to form the nucleosome that comprises the basic unit of chromatin [18] and thus functions as a surrogate cellular transcriptional unit. HTLV-1 exists primarily as a cell-associated provirus that is transmitted primarily through cell-to-cell contact [11] via a virological synapse [19], [20].

Gene expression following proviral integration is a crucial stage in the HTLV-1 retroviral life cycle, which depends on the viral transcriptional transactivating protein Tax [21], [22] and specific cellular transcription factors during both infection and viral reactivation from latency [23], [24]. The 40-kDa Tax protein is involved in upregulating HTLV-1 gene expression by interaction with three 21-base pair (bp) Tax-responsive elements (TRE) located within the U3 region of the viral promoter or long terminal repeat (LTR) [25], [26], [27]. Each TRE comprises domains A, B, and C [28] with the central B region consisting of a conserved 8-nucleotide core sequence that closely mimics a cyclic AMP-responsive element (CRE) and is flanked by 5′ and 3′ G/C rich sequences [29]. Tax activates transcription by interfacing with a number of cellular transcription factors: CRE binding protein (CREB) and serum response factor (or p67^SRF^) to the CRE [30], [31]. Tax interacts with dimeric CREB as a homodimer forming a ternary complex that in turn facilitates the stabilization of a CREB/TRE complex [29], [32]. Once stabilized, Tax independently recruits two cellular coactivators, p300/CREB-binding protein (p300/CBP) and p300/CBP-associated factor (P/CAF), both of which bind two distinct regions in the amino- and carboxyl-terminus of Tax, respectively, eventually activating transcription by histone acetylation through chromatin remodeling [33], [34], [35], [36]. In addition, Tax reduces histone protein and transcript levels in HTLV-1-infected compared to uninfected T-cell lines [37], [38].

However, most of the investigations highlighting the importance of the cellular transcription factors in HTLV-1 Tax-mediated LTR activation [30], [39], [40], [41], [42] and the ability of Tax protein to interact with these factors independently [33], [36], [43] have been performed using transiently transfected viral reporter plasmids or in cell lines that otherwise do not represent the primary target for HTLV-1 *in vivo*. Further studies with HIV-1 indicated that the integrated provirus differs from a transfected viral plasmid both physically [44] and in the requirement of certain cellular factors, especially those belonging to the chromatin remodeling histone acetyltransferase (HAT) family [45], [46], [47] or even the transcriptional repressor domain [48]. These results demonstrate that transient transfection studies may not convey the complete picture by undermining the crucial role of chromosomal structure in transcriptional regulation. Hence, to better understand viral gene regulation and the complex interplay between the integrated provirus, host cellular transcription factors, and the viral Tax protein during the course of infection and reactivation following latency, it is imperative that such studies be performed with the stably integrated viral promoter formatted in the context of cellular chromatin.

With respect to regulation of the HTLV-1 promoter, it has been demonstrated that indeed a differential requirement exists for the cellular transcription factors in the activation of stably integrated versus transiently transfected LTR [49]. Therefore, we decided to examine Tax-mediated regulation of an integrated HTLV-1 LTR. To this end, LTR stable integrants with a reporter luciferase gene (LTR-luc) were generated in the Jurkat cell line, representative of the natural target CD4^+^ T cells, to characterize the intricacies involved in the interplay between the integrated provirus, cellular transcription factors and Tax protein. Herein, we demonstrate the successful generation of Jurkat T-cell clones harboring stably integrated HTLV-1 LTR-luc. Further, to investigate the comparative activation/repression of cellular factors between stably integrated and transiently transfected LTR in the absence and presence of Tax, a high-throughput transcription factor analysis was performed using protein-DNA array technology. Many substrates and factors associated with the two major chromatin-remodeling complexes, SWI/SNF and HATs, were activated in the stably integrated clones following transfection with Tax. To explore the observed heightened activation of factors necessary for chromatin remodeling complexes, we explored the upstream microRNA (miRNA) regulatory pathway by miRNA microarray. Herein, a global downregulation in the expression of cellular miRNAs in the HTLV-1 LTR-luc stably integrated CD4^+^ T-cell clone was observed in the presence of Tax, implying the ability of Tax to modulate the cellular miRNA machinery. When compared to results presented with the transcription factor array, many of the downregulated miRNAs were found to target the mRNA coding for the P/CAF and p300 HAT family members, suggesting a role for Tax in downregulating the expression of cellular miRNAs that are in turn involved in suppressing the expression of factors involved in chromatin remodeling. The results presented herein demonstrate that the Tax protein can modulate the cellular miRNA machinery and downregulate the expression of miRNAs that could be involved in regulating the chromatin remodeling factors.

## Materials and Methods

### Cell culture

Jurkat CD4^+^ T cells (ATCC# TIB-152) and the HTLV-1-producing rat cell line TARL-2 [50] were propagated at 37°C with 5% CO_2_ and 90% relative humidity in RPMI-1640 (Mediatech, Herndon, VA) supplemented with penicillin (Mediatech, 100 U/ml), streptomycin (Mediatech, 100 µg/ml), sodium pyruvate (Mediatech, 1.0 mM), HEPES buffer (Mediatech, 10 mM), 0.45% glucose (Mediatech), and 10% fetal bovine serum (Thermo Fisher Scientific, Waltham, MA).

### Plasmids

The plasmids, pCMV-Tax and the pGL3-based HTLV-1 LTR luciferase reporter vector pU3R-luc, were kindly provided by Dr. Kuan-Teh Jeang (NIH, Bethesda, MD) and have been described previously [51]. The plasmids pGL3 basic and pUC18 and parental pCMV4 plasmids were obtained commercially (Promega, Madison, WI). The plasmid pRSV-neo encoding the selectable marker for neomycin resistance was obtained from ATCC (Manassas, VA; catalog #37198).

### Stable integration of HTLV-1 LTR in Jurkat cells

Jurkat cells were plated in 100-mm culture plates with antibiotic-free medium at a seeding density of 5×10^6^ cells per plate. Cells were co-transfected with 8 µg of pU3R-luc plasmid and 0.8 µg of pRSV-neo plasmid using Lipofectamine (Invitrogen, Carlsbad, CA). Twenty-four hours post-transfection, the cells were transferred to antibiotic-containing medium. On the third day, G418 (Geneticin; Invitrogen) was added to the cell culture at a concentration of 500 µg/ml. After 15 days of G418 selection, several single clones were isolated by performing serial dilutions and were propagated to confluence.

### Luciferase assay

Cell lysates were prepared from 1×10^6^ cells using 50 µl of 1×Passive Lysis Buffer (Promega, Madison, WI), and luciferase activity was assayed using the luciferase assay system (Promega) on the Glomax 96 microplate luminometer (Promega).

### Polymerase chain reaction

To verify integration of the HTLV-1 LTR-luc in the clones, polymerase chain reaction (PCR) was performed to detect both the LTR and luciferase gene using the following primer sequences: for (a) HTLV-1 LTR (F:5′GACCAAGGCTCTGACGTCTC3′; R:5′TTTTGAGGTGAGGGG TTGTC3′) and (b) luciferase open reading frame (F:5′ATGGAAGACGCCAAAAACATAAAG AAAGG3′; R:5′TAGAATTACACGGCGATCTTTCCGC3′) with amplicon sizes of 183 bp and 1.6 kb, respectively. The standard PCR reaction involved using 45 µl of PCR Supermix (Invitrogen), 10 µM each of forward and reverse primer, and 50 ng of DNA in a total volume of 50 µl. The thermal cycling conditions comprised an initial activation step at 95°C for 2 min followed by 35 cycles involving denaturation at 95°C for 30 sec, annealing at 54°C for 30 sec, extension at 72°C for 1 min, and a final extension at 72°C for 10 min.

### Southern blot hybridization

Genomic DNA was extracted from stably transfected Jurkat cells using the Wizard SV Genomic DNA extraction procedure (Promega), and 10 µg of the extracted DNA was digested overnight with *Eco*RI and amplified for the luciferse gene by PCR. Resolved PCR products were blotted onto a nylon membrane. The membrane was hybridized with a luciferase open reading frame fragment as probe and excised from the pGL3 vector using restriction enzymes *Nco*I and *Xba*I [49]. The probe was labeled with [α-^32^P] dCTP using the GE Amersham Rediprime II Labeling System (GE Healthcare Biosciences Corp, Piscataway, NJ). The following day, membranes were washed, screened, and scanned on Amersham Biosciences STORM 820 using the STORM scan software, and the pictures were analyzed using ImageQuant software (GE Healthcare Biosciences, Piscataway, NJ).

### Real-time PCR

To generate standard curves, DNA was extracted from the Jurkat cells for β-actin and TARL-2 for LTR. Because the single Jurkat cell has two copies of β-actin, we calculated that 1 ng of DNA contains 333 copies of the β-actin gene, whereas for TARL-2, which is an HTLV-1-infected cell line with a single copy of HTLV-1 proviral DNA [50], 1 ng of DNA contains 167 copies of LTR [52]. The extracted DNA samples were serially diluted to get 10^5^, 10^4^,10^3^, 10^2^, 10, and 1 copy for both β-actin and LTR. Real-time PCR was performed on an ABI Prism 7300 Sequence Detector (Applied Biosystems, Foster City, CA). The standard real-time PCR reaction using SYBR Green I consisted of 15 µl SYBR Green PCR Master Mix (Applied Biosystems), 10 µM each of forward and reverse primers (IDT, Coralville, IA), and 10 µl of DNA in a total volume of 30 µl. The thermal cycling conditions comprised an initial activation step at 95°C for 10 min followed by 40 cycles including denaturation at 95°C for 15 s and annealing and extension at 60°C for 1 min. The following primers were used for HTLV-1 LTR (F:5′GACCAAGGCTCTGACGTCTC3′; R:5′TTTTGAGGTGAGGGGTTGTC3′) and β-actin (F:5′CAGCGGAACCGCTCATTGCCAATGG3′; R:5′TCACCCACACTGTGCCCATCTACG A3′). Experiments were performed in duplicate for each data point. Dissociation or melting curve analysis was implemented to ensure the presence of a single peak at the correct melting temperature. The standard curves for β-actin and LTR were used with a coefficient of correlation, *r^2^*, being greater than 0.996 for both. From the standard curves, the C_T_ values for LTR and β-actin for different clones were obtained and used to calculate the copy number. Copy number per cell for each clone was calculated from the formula (copy number of LTR)/(copy number of actin/2)×10^2^, and expressed as the number of HTLV-1 integrated copies per 100 cells.

### Transient transfections

Five million cells per well of the selected clone were plated in a 100-mm tissue culture dish (BD Labware, Franklin, NJ) in antibiotic-free medium the day before transfection. Transient transfection was performed using FuGene 6 transfection reagent as described by the manufacturer (Roche, Indianapolis, IN). For clones with a stably integrated LTR, the transfection setup included 6 µg of pCMV4 (empty expression vector) or 1 µg of pCMV-Tax and 5 µg of pUC18. For cells with a transiently transfected LTR, the setup included 1 µg of pCMV4 and 5 µg of pU3R-luc or 1 µg of pCMV-Tax and 5 µg of pU3R-luc. The indicated amounts of pUC18 plasmid DNA were used to obtain an equal amount of total DNA in each experimental design. Cells were harvested 24 h post-transfection and processed to obtain cell extracts (luciferase assay), nuclear extracts (transcription factor array), and total miRNAs (miRNA microarray).

### Protein-DNA transcription factor array

Nuclear extracts were isolated from 1×10^6^ transfected cells as described by the manufacturer (Panomics, Fremont, CA) and the protein content was determined using the Bradford assay (Bio-Rad Laboratories, Hercules, CA). The protein-DNA array membranes (PD Array I, Panomics) were pretreated at 42°C overnight with hybridization buffer. The nuclear extract was labeled with the TranSignal Probe mix as described by the manufacturer (Panomics). The labeled probe was eluted and hybridized to the array membranes. The bound probe was detected by treating with streptavidin-horseradish peroxidase substrate after hybridization. The membranes were then exposed using the chemiluminescence imaging system (Fluorchem Imager, Cell Biosciences, Santa Clara, CA), and the spots were analyzed using ImageQuant software (GE Healthcare Biosciences). Spots from each membrane were normalized to their respective positive controls after background subtraction.

### miRNA isolation, labeling, and hybridization

Total miRNA was isolated as described by the manufacturer using the PureLink miRNA isolation procedure (Invitrogen). After the miRNA was quantitated, poly (A) tail was added to the miRNA followed by ligation to a DNA polymer already labeled with Alexa Fluor dye using the NCode miRNA Rapid Labeling module (Invitrogen). Stably integrated clone C5 transfected with pCMV-Tax control was labeled with Cy5 (Alexa Fluor 635), and the clone C5 transfected with pCMV was labeled with Cy3 (Alexa Fluor 532). The purified tagged miRNA was hybridized to an NCode Human miRNA Microarray V3 (Invitrogen), which is an epoxy-coated glass slide printed with miRNA probes targeting all of the known human miRNAs in the miRBase Sequence database, release 10.0, in an antisense orientation. Following overnight hybridization, the array was washed. Within 30 min of the final wash, the array was scanned and analyzed on the microarray scanner Genepix 4000A (Axon Instruments, Molecular Devices, Sunnyvale, CA) at 635 nm and 532 nm.

### Validation of miRNA array data and confirmation of target mRNAs

From the miRNA array data, miR-149 and miR-873, that were found to be down regulated by 0.2-fold, were selected and subjected to further confirmation in an HTLV-1-infected T cells line MT-2. Total RNA was extracted from by using the TRIzol reagent (Life Technologies) and subjected to cDNA preparation using the TaqMan MicroRNA RT kit (Applied Biosystems). The standard real time PCR reaction was set up using RT primers specific for miR-135b, miR-149 or miR-873 provided in the microRNA assay. The thermal cycling conditions comprised an initial activation step at 95°C for 10 min followed by 40 cycles including denaturation at 95°C for 15 s and annealing and extension at 60°C for 1 min.

Following confirmation of miRNA presence in Jurkat cells and their downregulation in MT-2 cells, specific miRNA mimics (200 pg) were transfected in these cells using Lipofectamine as per manufacturer's instruction (Invitrogen). Thirty-six hour post-transfection, one half of the transfected cells was harvested and processed to obtain total cell lysates using M-PER Mammalian Protein Extraction Reagent (Pierce, USA). The other half of the cells were washed and resuspended in fresh medium. After additional 2–3 days, supernatants were collected to carry out HTLV-1 p19 ELISA (ZeptoMetrix Corp., Buffalo, NY, USA) in order to assess the levels of viral progeny production. Western blot analysis was performed to assess the effect of miRNA mimics on target mRNAs (p300 and p/CAF). Total cell lysates were resolved by SDS-PAGE and transferred to a PVDF membrane. The blots were blocked for 1 h with Odyssey blocking buffer (Licor, Lincoln, USA), and rinsed with PBST (PBS+0.05% Tween-20, once for 10 min and twice for 5 min). The membranes were then incubated with anti-p300 and anti-p/CAF monoclonal antibodies (each 1∶1000 dilution, Abcam, Cambridge, USA) for 2 h at room temperature and rinsed with PBST as before followed by 1 h incubation with an IRDy800-conjugated anti-rabbit and IRDy680-conjugated anti-mouse secondary antibody, respectively. Signals were detected by Licor Pearl Pulse Imager.

## Results

### Construction of CD4^+^ T-cell line with stably integrated HTLV-1 LTR-luc

Most previously published studies concerning the regulation of the HTLV-1 promoter have relied on transient transfection of LTR-containing plasmids into a variety of different target cells. The structural conformation, basal transcriptional activity, and requirement for cellular transcription factors with regard to the integrated chromatin-associated HTLV-1 LTR are different from a transiently introduced LTR plasmid as observed in CHOK1 and HeLa cell types [49]. Moreover, each cell type has different endogenous levels of transcription factors, thereby introducing another variable [49]. Therefore, to better understand the regulation of the viral promoter when integrated into the genome of the clinically relevant CD4^+^ T cell, we constructed a stably integrated HTLV-1 LTR-luc reporter in the CD4^+^ T-cell line, Jurkat.

**Figure 1:**
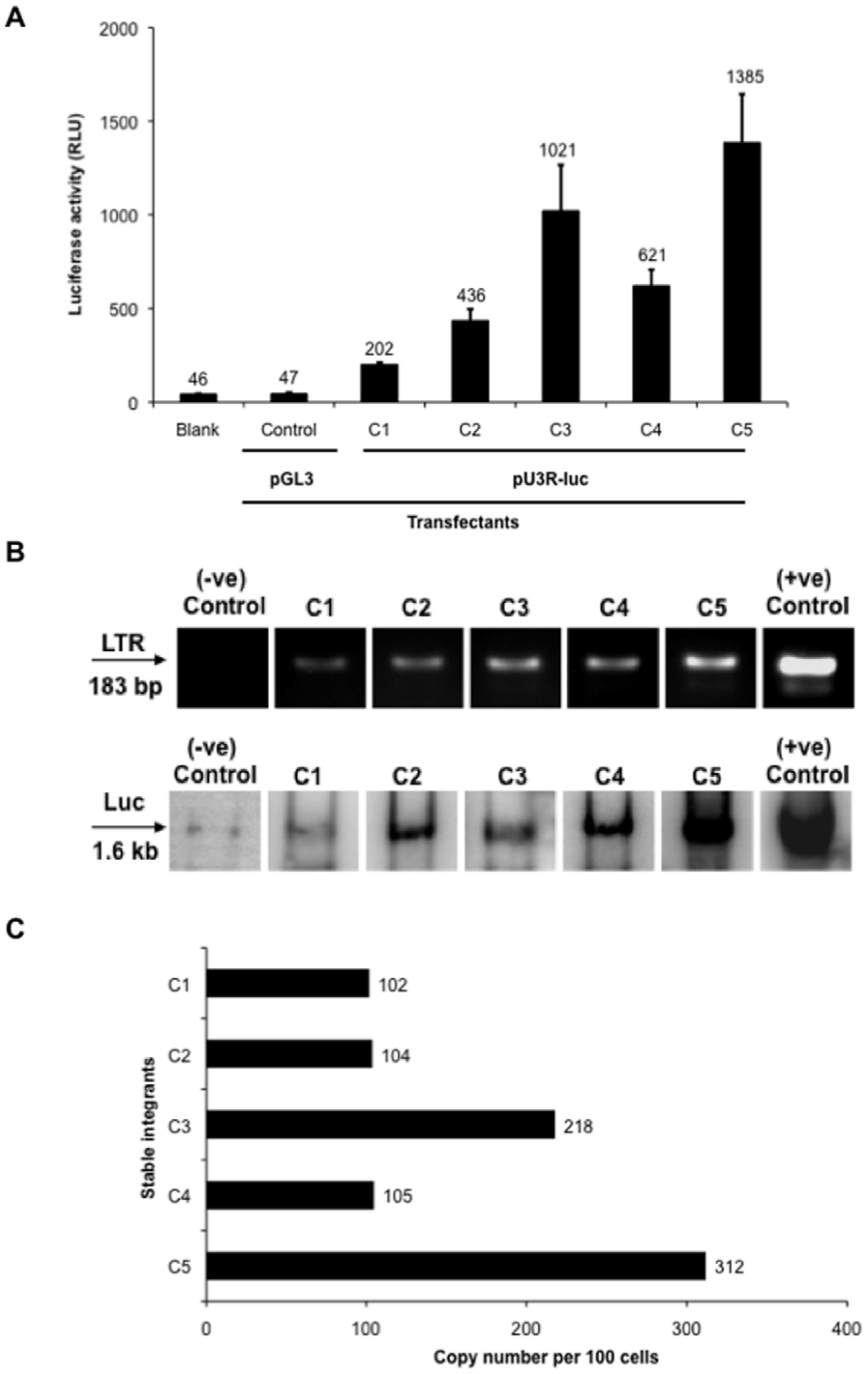
Screening of HTLV-1 LTR-luc stable integrants. (A) Luciferase activity in stably integrated HTLV-1 LTR Jurkat clones was measured in terms of relative light units. Control represents the luciferase activity from the cells that were stably integrated with pGL3-basic vector and pRSV-neo. Each value shown represents the average of duplicate transfection reactions and is representative of at least two independent experiments. Error bars indicate standard deviation (±) from one representative experiment. (B) For verifying HTLV-1 LTR-luc integration, all five clones (C1–C5) were examined and confirmed qualitatively for HTLV-1 LTR-luc integration for both LTR and luciferase genes independently by PCR amplification of LTR with product size 183 bp and Southern blot hybridization using luciferase probe following PCR amplification of luciferase gene with product size 1.6 kb. The control clone represents the Jurkat clone stably integrated with pGL3-basic vector and pRSV-neo in the absence of the HTLV-1 LTR. The positive control in each case is the pU3R-luc plasmid. (D) For quantifying HTLV-1 provirus copy numbers by real-time PCR in the stably integrated clones, all five clones (C1–C5) were selected for real-time PCR analysis to determine the copy number of copies of the integrated LTR per 100 cells. Standard curves for β-actin and the HTLV-1 LTR were generated from Jurkat and TARL-2 cell lines, respectively, with the coefficient of correlation, *r^2^*, being greater than 0.996 for both.

The Jurkat CD4^+^ T cells were cotransfected with the HTLV-1 LTR-luc (pU3R-luc)- and the antibiotic selection marker (pRSV-neo)-containing plasmids using the Lipofectamine transfection reagent and then selected for G418 (Geneticin) resistance for 15 days. A stably integrated control clone (without the HTLV-1 LTR) was also derived by co-transfecting Jurkat cells with the pGL3-basic and pRSV-neo plasmids. After the selection period, 25 clones of Jurkat CD4^+^ T cells were isolated by serial dilution and screened for integrated pU3R-luc with five representative clones selected for further analysis. As shown in Fig. 1, different levels of basal luciferase expression were observed across the five representative clones in the initial screening process with expression values ranging from lowest (202 relative light units for clone 1) to highest (1385 relative light units for clone 5). The wide range in the luciferase expression was likely due to the different sites of integration of the HTLV-1 LTR-luc in the host genome. Previous studies have shown that integration into a transcriptionally active site will allow for a greater LTR-mediated transcription of the *luc* gene with a higher luciferase expression compared to the integration in a transcriptionally inactive region [53], [54], [55]. The control clone shown in Fig. 1A represents luciferase expression from the cell lysate of a Jurkat control clone, which has the luciferase open reading frame but no promoter region to drive the luciferase expression. All other individual stably integrated Jurkat clones were propagated separately to maintain long-term homogeneity. To rule out the possibility of a mutation within the integrated viral promoter being responsible for the observed variation in the LTR activity in the clones, the five clones were PCR-amplified using the primers RV3 and GL2 with specificity for the insert (integrated HTLV-1 LTR-luc) within the multiple cloning site of the pGL3 vector and sequenced (GENEWIZ, Inc, South Plainfield, NJ). The sequence for each clone was then compared to the original LTR sequence within the known HTLV-1 genome (NCBI accession number AF139170) [56]. The sequencing results demonstrated complete similarity with regard to the integrated HTLV-1 LTR-luc sequences from all five clones as compared to the original LTR sequence, thus eliminating the possibility of any mutation within the LTR being responsible for the observed differences in luciferase expression (data not shown). The analysis of clones with luciferase expression values below or equal to those of the control was discontinued.

After selecting the G418-resistant and luciferase-expressing clones, HTLV-1 LTR-luc integration into the genome of the Jurkat clones was confirmed both qualitatively and quantitatively. Qualitative verification was performed with all five clones by PCR using the HTLV-1 LTR primers (product size 183 bps) confirming its presence (Fig. 1B). Similar results were obtained when Southern blot hybridization was performed on the PCR-amplified luciferase product using a luciferase probe, with a stronger signal for clone C5, whereas a weaker signal was observed for the remaining clones (Fig. 1B). These results clearly indicate the presence of an integrated HTLV-1 LTR-luc in all five clones by verifying independently the presence of both LTR and luciferase genes. For quantitative assessment, real-time PCR was performed to calculate copy numbers of the integrated HTLV-1 LTR in all five clones after generating standard curves for both β-actin and LTR using both the Jurkat and TARL-2 cell lines, respectively. As shown in Fig. 1C, approximately two and three copies of the integrated provirus per cell were detected in clones C3 and C5, respectively. The remaining clones (C1, C2, and C4) had at least 1 copy per cell (Fig. 1C). Overall, the results to date indicate that the Jurkat clones selected after generation of stable integrants demonstrate successful integration of the HTLV-1 LTR-luc. Unlike the many copies of HTLV-1 LTR integrants observed in CHOK1 and HeLa [49], the results obtained with Jurkat cells were probably different because the transfection efficiency of Jurkat cells is much lower than that of either CHOK1 or HeLa. The results obtained with the HTLV-1 LTR Jurkat cell clones were more similar to those obtained with infected T-cell clones examined *ex vivo* that preferentially contain a singly integrated provirus [57]. Moreover, asymptomatic HTLV-1-infected individuals have low proviral loads (2–3 copies per 100 cells) in peripheral blood mononuclear cells, suggesting low integration efficiency in T cells [58], [59], [60]. Based on the results obtained and the fact that infected T-cell clones have a low proviral DNA load, experimental attention was focused on clones 1, 2, and 4. We pooled the selected clones (clones 1, 2, and 4), thereby generating a mixed population of stably HTLV-1 LTR transfected CD4^+^ T cells, representative of the infected cell populations found *in vivo*. All subsequent transfection, transcription factor profiling, and miRNA array analyses were performed using this mixed pool of selected clones.

### Effect of Tax on the integrated HTLV-1 LTR activity and cellular transcription factors

**Figure 2:**
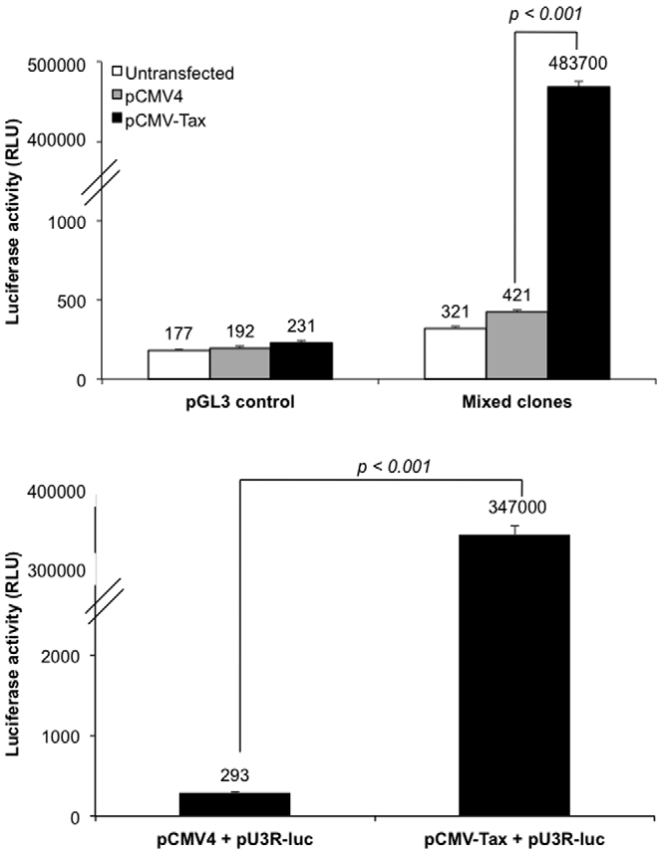
Verifying the functionality of the integrated HTLV-1 LTR-luc by determining the ability to respond to Tax. The stably integrated pooled clone (clones C1, C2, and C4) was compared to transiently transfected HTLV-1 LTR-luc Jurkat with respect to its ability to respond to Tax by transiently transfecting both cell lines with either pCMV (control) or pCMV-Tax. Luciferase activity was measured 24 h post-transfection. (A) Stably integrated pooled clone versus the control clone (without integrated viral promoter). (B) Transiently transfected Jurkat clone with pU3R-luc versus control Jurkat (without transfected viral promoter). Each value shown represents the average of duplicate transfection reactions and is representative of two independent experiments. Error bars indicate the standard deviation of one representative experiment.

To perform a comparative analysis and better understand the regulation of the stably integrated versus transiently transfected HTLV-1 LTR in the absence and presence of Tax in the context of cellular transcription factors representative of the natural host cell target, the CD4^+^ T cell, we performed transient transfections with a Tax-expressing plasmid in the pooled stably integrated Jurkat cell clones and co-transfections with both HTLV-1 LTR and Tax-expressing plasmids in parental Jurkat cells. The stably integrated pooled Jurkat mixed cell clones were transiently transfected with either pCMV4 (empty expression vector) or pCMV-Tax (Tax expression vector), whereas Jurkat was transiently transfected with either pU3R-luc (HTLV-1 LTR expression vector) or co-transfected with pU3R-luc and pCMV-Tax. All transfectants were harvested independently after 24 h, and the cellular (for luciferase assay) and nuclear (for transcription factor analyses) extracts were processed as described. The luciferase expression from the cell lysate of pooled stably HTLV-1 LTR integrated Jurkat T-cell clones (Fig. 2A) and the transiently transfected parental Jurkat cell line (Fig. 2B) indicate approximately a 1000-fold significant increase (p<0.001) in expression of the stably integrated reporter luciferase gene when transfected with Tax (Fig. 2A), implying the ability of Tax to activate and drive luciferase expression via the integrated viral promoter. This finding also validates the functional status of the integrated viral promoter. Similarly, we noted a significant increase (p<0.001) in luciferase expression with the transiently transfected viral promoter when transfected with Tax.

**Figure 3:**
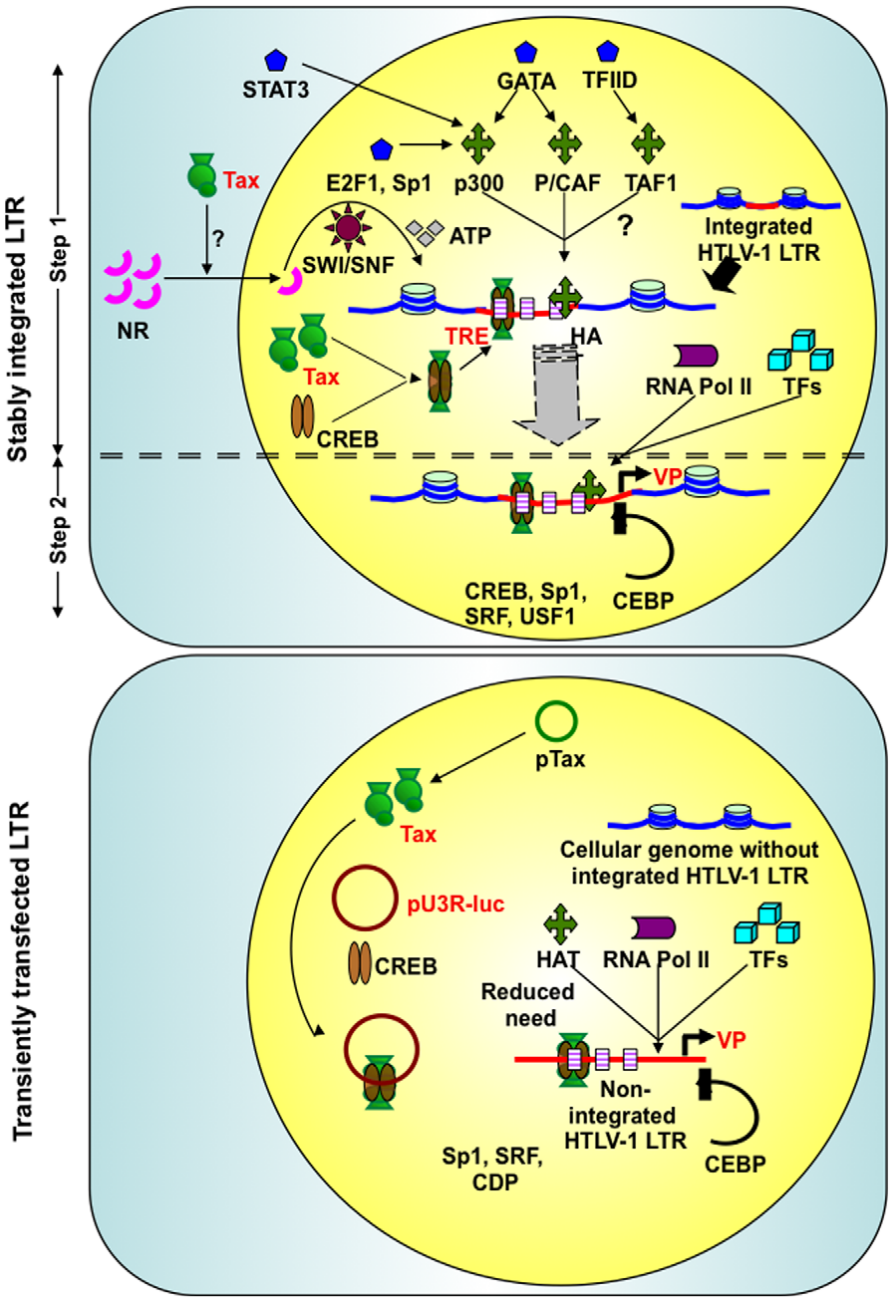
Mechanism to explain the differential requirements of cellular chromatin remodeling factors in stably integrated versus transiently transfected HTLV-1 LTR. The major differences in the need for cellular chromatin remodeling factors between a stably integrated and transiently transfected viral promoter are outlined. In the case of a stably integrated LTR, Tax mediates the increased recruitment of the chromatin remodeling factors (HATs and SWI/SNF) and their substrates. In a transient transfection, the LTR is more readily accessible and thus exhibits a reduced need for such factors.

Tax is involved in the most critical step in retroviral replication that can lead either to viral replication and proliferation or to a state of dormancy/latency. To evaluate the impact of Tax on cellular transcription factors and signaling pathways in the newly generated HTLV-1 LTR stable T-cell integrant versus a T cell transiently transfected with the HTLV-1 LTR, we performed high-throughput analysis on various cellular transcription factors. We used a DNA-protein array that offered the advantage of analyzing multiple transcription factors at once as opposed to individual factors by electrophoretic mobility shift assays. The transcription factors associated with both stably integrated and transiently transfected LTRs, which are characteristic of the major pathways involved in HTLV-1 pathogenesis, were identified, and their relative levels were analyzed. Many of the major factors (CREB, Sp1, C/EBP, and NFAT), their interacting proteins as well as those involved in the JAK/STAT and TGF-β signaling pathways, were either activated or repressed following Tax expression (Table 1). The factors STAT3 and SMAD3/4 were activated in stable integrants, implying involvement of both the JAK/STAT and TGF-β pathways. Because a stably integrated LTR would have a preferential requirement for factors involved in chromatin remodeling, the substrates (E2F1, GATA, and TFIID) and factors (members of nuclear receptor family) for the two main groups of chromatin remodeling complexes, HAT and SWI/SNF, respectively, were highly activated in the stable integrants. Figure 3 explains and compares the importance of chromatin remodeling machinery under stably integrated and transiently transfected HTLV-1 LTR setting. It also highlights potential new players in this process. As projected, there is increased need for chromatin remodeling factors and their substrates in stably integrated LTR. However, in case of transiently transfected LTR, the need for such factors is reduced. The entire data from this analysis has been deposited into the GEO database with an accession number “GSE33628”.

**Table 1:** Transcription factor profiling of T cells containing stably integrated or transiently transfected HTLV-1 LTR.

	Transcription Factor		Fold Change (pCMV-Tax/pCMV)	
			Stable	Transient
**Nuclear Factors**	**Upregulated**			
	E2F1*	E2F transcription factor 1 (substrate for p300)	2.4	0.7
	GATA*	GATA binding protein (substrate for p300 and P/CAF)	30	54
	TFIID*	Transcription factor II D (substrate for TAF1)	4.1	0.7
	CREB	cAMP responsive element binding protein	8.2	0.2
	NF-E1	Nuclear factor (erythroid derived 1)	5.4	0.9
	NF-E2	Nuclear factor (erythroid derived 2)	2.7	1.1
	SMAD3/4	Small mothers against decapentaplegic 3/4	2.1	1.1
	SP1	Stimulatory protein 1	15	1.2
	SRE	Serum responsive element	3.1	0.7
	USF1	Upstream stimulatory factor 1	3.5	0.7
	**Downregulated**			
	AP1	Activator protein	<0.1	<0.1
	BRN3	POU class 4 homeobox 2	0.1	0.7
	CDP	CCAAT displacement protein	<0.1	40
	CEBP	CCAAT enhancer binding protein	<0.1	<0.1
	c-Myb	v-myb myeloblastosis viral oncogene homolog (avian)	<0.1	<0.1
**Cytoplasmic Factors**	**Upregulated**			
	GR**	Glucorticoid receptor	3.5	0.9
	PPAR**	Peroxisome proliferator activated receptor	8.4	29
	RAR**	Retinoic acid receptor	3.4	1.5
	RXR**	Retinoid X receptor	3.7	1.5
	VDR**	Vitamin D receptor	3.2	0.3
	NFAT	Nucelar factor of activated T cells	41	1.8
	STAT1	Signal transducer and activator of transcription 1	5.2	2.1
	STAT3	Signal transducer and activator of transcription 3	3.7	0.3
	**Downregulated**			
	PBX	PBX knotted 1 homeobox 2	0.1	0.5

* **Substrates for histone acetyltransferases (HATs) - p300, P/CAF, TAF1.**

** **Nuclear receptor (NR) family.**

List of various cellular transcription factors that were differentially up- or downregulated in case of stably integrated or transiently transfected viral LTR.

### miRNA profiling of Jurkat with stably integrated LTR in the presence of Tax

Because of these compelling observations, we examined the role of the Tax protein on the host miRNA machinery. The hypothesis was that if Tax can modulate the host cellular miRNA machinery, it should be able to downregulate the expression of those miRNAs that are involved in repressing the mRNA for members of the chromatin remodeling family. Based on results involving the increased activation of chromatin remodeling substrates and factors (Table 1), we theorized that the Tax protein could act upstream by regulating the expression of miRNAs that target mRNAs destined to be translated into chromatin remodeling factors. This hypothesis was strengthened by a study highlighting the ability of HIV-1 to suppress the expression of a miRNA linked to P/CAF during viral replication [61].

**Figure 4:**
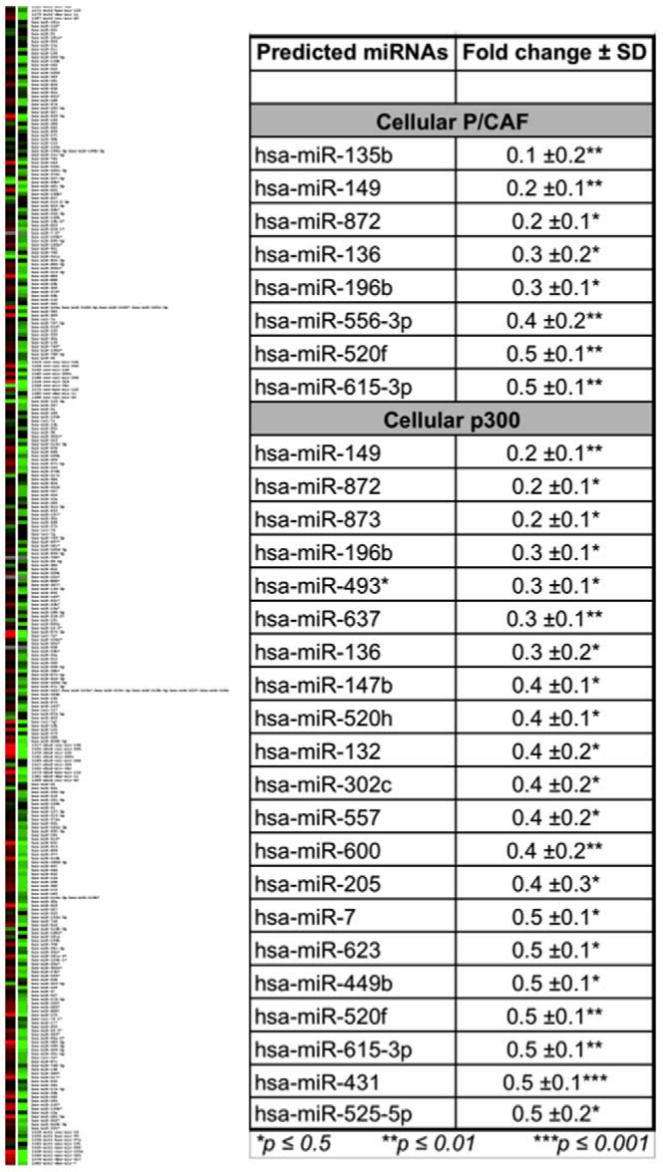
Ability of HTLV-1 Tax protein to modulate and thus suppress the expression of cellular miRNAs. A miRNA microarray cluster was used to demonstrate the ability of HTLV-1 Tax to globally downregulate the expression of cellular miRNAs. The two lanes represent the Cy5/Cy3 ratio in dye flip. The miRNAs isolated from the stably integrated pooled Jurkat clone transfected with Tax were labeled with Cy5 whereas those isolated from the same integrated clone but transfected with control plasmid (pCMV4) were labeled with Cy3. The table shows the high probability miRNAs against P/CAF and p300 that have been downregulated in the presence of HTLV-1 Tax.

A miRNA microarray was performed to determine if HTLV-1 Tax can modulate the cellular miRNA machinery and result in the suppression of miRNAs linked to the chromatin remodeling enzymes (P/CAF and p300). The miRNAs were isolated 24 h after transfection of stably integrated pooled clones that were either transfected with pCMV-Tax or the empty plasmid pCMV (control). Each microarray experiment was performed in duplicate, and only miRNAs that were consistently upregulated/downregulated in both slides were examined. The miRNAs that displayed changes in expression levels by more than 2.0 or less than 0.5 were short-listed for further analysis. The fold-change is derived from the ratio of means for Cy5/Cy3, where cell lines with stably integrated LTR transfected with pCMV-Tax were labeled with Cy5 whereas those transfected with pCMV were labeled with Cy3. Analysis showed that Tax globally downregulated the expression of cellular miRNAs in Jurkat carrying the stably integrated HTLV-1 LTR-luc (Fig. 4). Overall, the expression level of 41 miRNAs was altered: 35 miRNAs were downregulated whereas six were upregulated after the introduction of the HTLV-1 transactivating protein Tax into the system that had a stably integrated LTR (Fig. 4). This initial observation suggested that Tax does indeed modulate the miRNA machinery in some manner. The next question was whether any of the downregulated miRNAs were involved in the regulation of the HATs. Using the Web-based tool MicroInspector [62], high-probability human miRNAs that target P/CAF and p300 were screened with free energy less than −20 kcal/mol at a hybridization temperature of 37°C. Of these, we found that eight miRNAs linked to the regulation of P/CAF and 21 miRNAs linked to the regulation of p300 were downregulated. These results corroborate the hypothesis that Tax does indeed interact with the cellular miRNA machinery to downregulate the expression of miRNAs that are in turn involved in the regulation of HATs, so as to enable the virus for transcribing viral proteins from the integrated LTR.

### Validation of miR-149 and miR-873 involvement in targeting p300 and p/CAF

**Figure 5:**
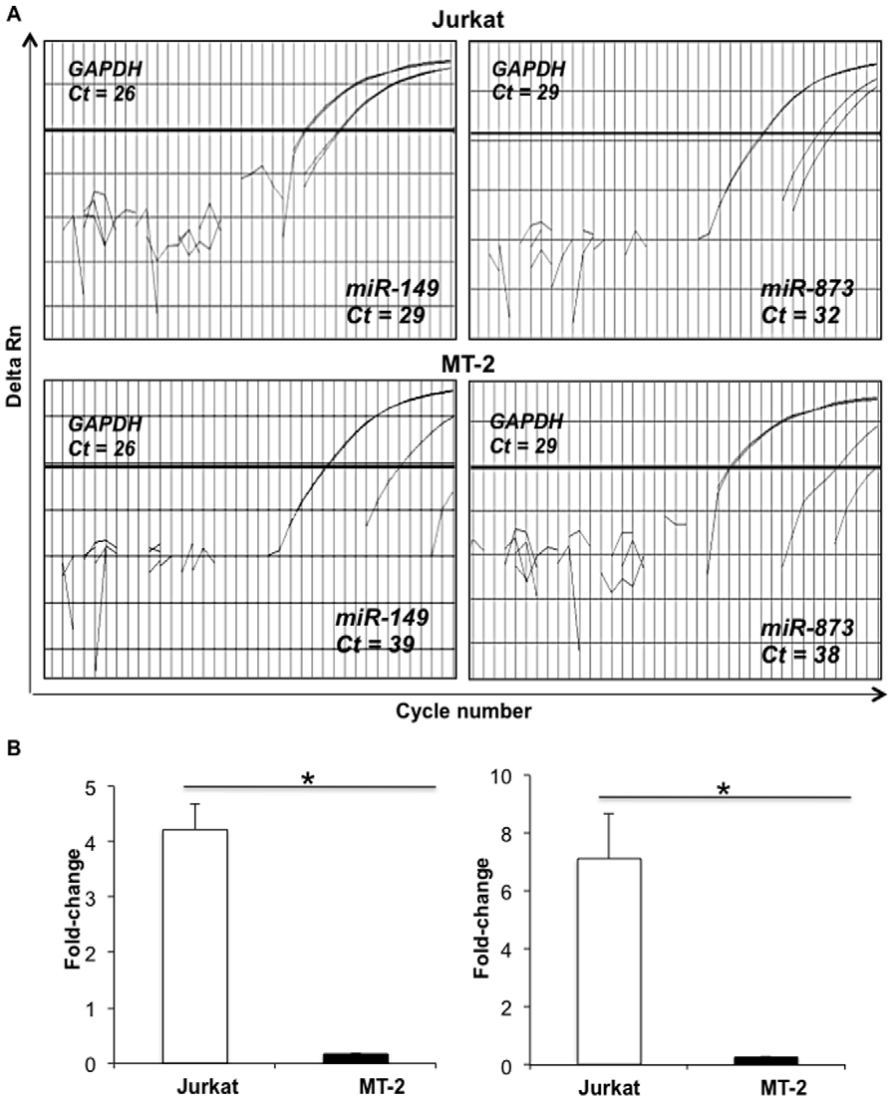
Down regulation of miR-149 and miR-873 in HTLV-1 infected cell line, MT-2. miR-873 were validated for their presence in MT-2 and Jurkat cells by real time PCR. Amplification plots were obtained for the same and plotted with their respective Ct values in both the cell lines with GAPDH as internal control (A). Fold-change in the expression was calculated based on the ΔΔCt method. Triplicate samples were utilized to derive the standard deviation and significance by t-test represented here as * for p<0.05.

For further analysis, we selected miR-149 and miR-873 that showed 5-fold down regulation and could potentially target both p300 and p/CAF (Fig. 4). Real-time PCR analysis specific to these microRNAs was performed to detect and compare their expression in control Jurkat T cell line cells as well as in active virus producing cell line, MT-2. Amplification profiles of both miRNAs clearly indicated their down regulation in MT-2 cells confirming our miRNA array results (Fig. 5A). While calculating the fold-change in expression by ΔΔCt method, miR-149 showed approximately 7-fold and miR-873 showed 5-fold down regulation in MT-2 cells as compared to Jurkat (Fig. 5B) further strengthening our microarray studies on clones.

**Figure 6:**
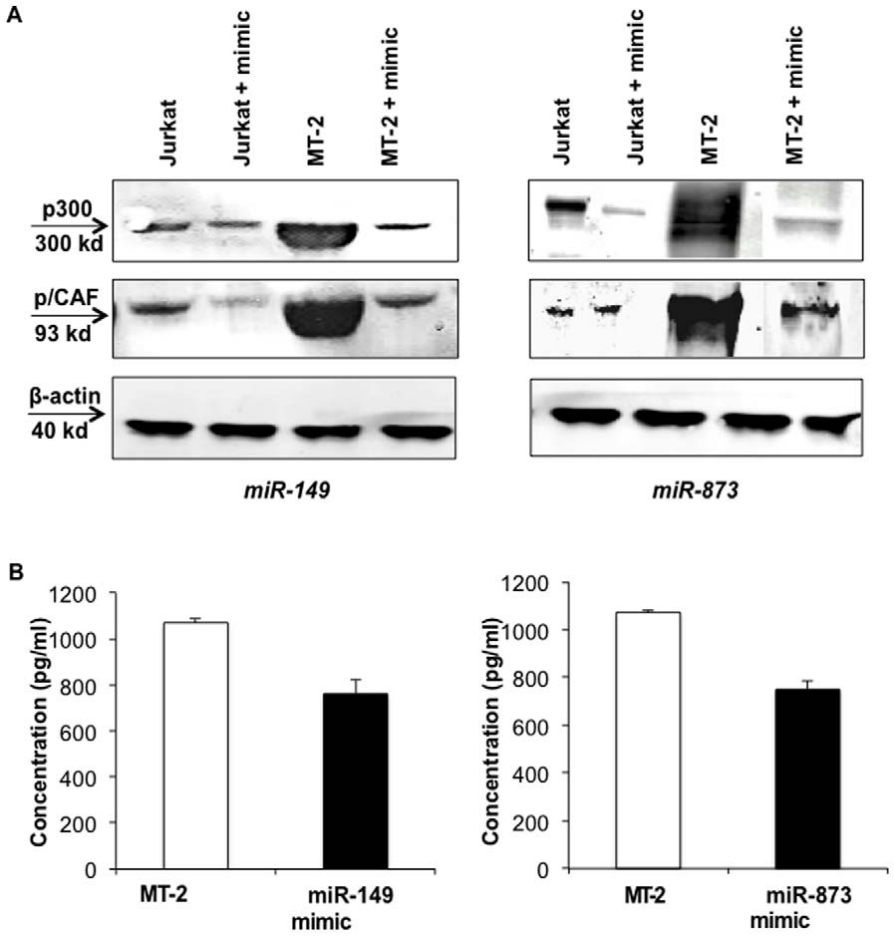
Effect of miRNA mimics on the expression of p300 and p/CAF in MT-2 cells. (A) Equal protein amounts from the whole cell lysates of MT-2 and Jurkat cells lines were resolved on SDS polyacrylamide gel and then transferred to a PVDF membrane. The membrane was then incubated with anti-p300 (1∶1000 dilution) and anti-p/CAF (1∶1000 dilution) monoclonal antibodies for 2 h at room temperature and incubated for 1 h with an anti-rabbit secondary antibody conjugated to IRDy800 and IRDy680, respectively. Signals were detected by Licor Pearl Pulse Imager. It was observed that the levels of p300 and p/CAF went down on transfection of MT-2 cells with respective miR-mimic when compared to non-transfected MT-2 cells. (B) HTLV-1 p19 was quantitated with ELISA in MT-2 culture supernatant and compared to supernatant obtained from MT-2 cells transfected with miR-149 and miR-873 mimics.

The next question was whether miR-149 and miR-873 were directly involved in regulating the HATs - p300 and p/CAF thereby controlling chromatin remodeling in HTLV-1 infected cells and aiding in viral replication. To test this, mimics of these microRNAs were transfected in MT-2 and Jurkat cells and their effect on the expression of putative target proteins was checked. Also, MT-2 cell culture supernatant was scored for viral protein (p19) production response prior to and after transfection. It was observed in western blot analysis that p300 and p/CAF protein levels were up regulated in MT-2 cells when compared with Jurkat cells but were sharply reduced in the presence of miRNA mimics (Fig. 6A). Jurkat cells showed only a slight down regulation in the levels of p300 and p/CAF in response to mimics since the levels of these proteins were already so low in these cells. With respect to viral progeny production in the presence of mimics, supernatants of MT-2 cells showed a drop in the level of viral protein p19 upon transfection. Overall, these results confirmed the array data and validated our hypothesis.

## Discussion

Viral latency and reactivation are essential phases of the retroviral life cycle that contribute to the persistence and pathogenesis mediated by the virus. Although latency has been well characterized in HIV-1 [63], much remains to be understood with regard to HTLV-1. Previous studies to characterize the mechanistic functioning of the viral promoter and its transactivating protein Tax have relied heavily on two major shortcomings: first, the use of transient transfection studies to examine the functional properties of the viral LTR, and second, the use of cell lines that are not representative of the primary target cell population in stable expression studies. A number of studies have shown that activation of the chromosomally integrated viral promoter is different from that of the transiently transfected promoter for both HIV-1 and HTLV-1 [44], [49]. Moreover, because each cell type differs in its endogenous levels of various cellular factors, it is important to compare relevant cell populations to be able to accurately identify and elucidate the role of different factors involved in the complex interplay between the virus and host cell.

To address the impact of cellular chromatin on LTR-directed viral transcription, we incorporated a stably integrated HTLV-1 LTR in the Jurkat cell line, which is representative of the clinically relevant target CD4^+^ T-cell population. Qualitative and quantitative assessments were used to screen, verify, and select clones from the initial population of stable integrants. Once the clones were screened, they were subjected to further analyses involving transcription factor and miRNA profiling, both in the absence and presence of Tax, to identify transcription factors involved in viral transcription and chromatin remodeling in the CD4^+^ T-cell population and the cellular miRNAs that might be differentially modulated by Tax in the context of an integrated viral promoter based on previous observations that each cell type is known to have its own repertoire of miRNAs [64]. Therefore, this study offers new insight into the regulation of the stably integrated HTLV-1 LTR and a new perspective on the impact of Tax on cellular miRNAs involved in the regulation of HATs in the primary cell phenotype targeted by HTLV-1 during the course of viral disease.

The transcription factor profiling following transfection with Tax in stably integrated LTR versus transiently transfected LTR cells yielded a number of expected results as well as some new observations (Table 1). The relative levels of transcription factors previously associated with HTLV-1-infected cells were highly activated (CREB, Sp1, SRE, NFAT, STAT1, and SMAD3/4) or repressed (C/EBP) in the stable HTLV-1 LTR-containing integrants compared to the transient transfections, highlighting the importance for the recruitment of such factors in the context of a stably integrated LTR. Factors not previously characterized in HTLV-1 LTR activation (BRN3 and PBX) had consistent levels in both stable and transient LTR cells. In addition, some factors that were highly activated (USF1, NF-E1, and NF-E2) or repressed (CDP) in the stably integrated LTR have not been previously documented to be involved in HTLV-1 infection. These factors may be involved in HTLV-1 transcription but may have been overlooked in earlier studies. The nuclear factors caught our attention: E2F transcription factor 1 (E2F1), GATA binding protein (GATA), and transcription factor IID (TFIID), as did the cytoplasmic nuclear receptor (NR) family factors: glucocorticoid receptor (GR), peroxisome proliferator-activated receptor (PPAR), retinoic acid receptor (RAR), retinoid X receptor (RXR), and vitamin D receptor (VDR). These factors serve as substrates or factors for the chromatin remodeling complexes: the HATs and SWI/SNF complexes, respectively [65]. However, two particular factors (GATA and PPAR) were found at reduced levels in stable compared to transient expression environments. Although the Tax protein has been shown to repress transcription mediated by certain members of the NR family (GR, RXR, and PPAR) in HeLa and CV1 cells [66], the results obtained in this study conflicted with the observed reduction in PPAR activation. From the transcription factor analyses (Table 1), we observed marked increases in the levels of members of the NR family. The NR family is a group of ligand-activated transcription factors that are located in inactive forms in the cytoplasm as part of a complex with heat shock proteins [67]. Upon ligand binding, these receptors dissociate from the complex and translocate to the nucleus where they function as transcription factors [67]. The NR family members GR, RAR, RXR, and VDR were upregulated in the Tax-transfected stable LTR integrants (Table 1). In fact, the GR and VDR promote remodeling activity by interacting with subunits of the SWI/SNF complex [68], [69].

RNAi is a cellular mechanism to silence gene expression and is predominantly mediated by miRNAs that target messenger RNA. The miRNAs are small endogenous RNAs (21–25 nucleotides) that regulate cellular gene expression at the posttranscriptional level, either by suppressing translation without affecting the stability of the mRNA as in invertebrate animals or by mRNA cleavage as mediated in plants [70]. Recent studies indicate the ability of retroviruses to interfere with the cellular miRNA regulatory pathway through the action of their transcriptional transactivating protein [71], [72] that can function as a suppressor of RNA silencing. Viruses can manipulate the cellular processes necessary for their replication and infection by targeting the host RNAi machinery. The expression profile of miRNAs in HIV-1-transfected human cells differs from that of control transfected cells [73], implying the modulation of this pathway by viruses. Bioinformatic tools have also highlighted the ability of inherent human miRNAs to target many viruses including HIV-1 and HTLV-1 [74]. However, as a counterbalance to the excitement generated by studies elucidating the role of viral transactivators in modulating host cellular miRNAs, some studies suggest otherwise [75]. An miRNA expression profiling of HTLV-1-transformed T-cell lines and primary peripheral blood mononuclear cells from ATL patients found six miRNAs that were constantly upregulated [76], two of which (miR-93 and miR-130b) targeted the 3′ UTR of the mRNA coding for a tumor suppressor protein, tumor protein 53-induced nuclear protein 1. Other studies have found several cancer-linked miRNAs (miRs 21, 24, 146a, 155, and 223) to be deregulated in HTLV-1 transformed cells [77]. All of these miRNAs were upregulated except miR-223, which was downregulated. Moreover, the expression of miR-146a was directly stimulated by Tax via NF-κB-mediated transactivation of its promoter. Analysis of hematopoietic-specific miRNAs found an aberrant expression of miRs (223, 181a, 150, 142.3p, and 155) in HTLV-1-infected cells *in vitro* and uncultured *ex vivo* ATL cells as well as altered expression of miRNAs involved in innate immunity regulation, particularly miRs (155, 125a, 132, and 146) [78]. On comparing the expression of miR-146a in HTLV-1-infected and -uninfected T-cell lines, Tax was found to induce the expression of miR-146a in a NF-κB-dependent manner and inhibited the expression of a gene harboring the target sequence of miR-146a on its 3′ UTR [79].

The miRNAs, like transcription factors, play a key role in the regulatory events of the cell; however, unlike transcription factors that can either positively or negatively regulate transcription, miRNAs primarily work through repression. Moreover, the miRNAs themselves are involved in regulating the expression of many transcription factors [80]. The role of miRNAs in chromatin remodeling, particularly in T-cell development and function, is a current area of investigation [81]. Therefore, the miRNA pathway serves as an ideal target for viruses that wish to control the regulation of many crucial proteins intrinsic to viral replication and survival as well as those that mediate antiviral effects. It is thus not surprising that current studies are centered on the mechanism by which retroviruses hijack and usurp the host miRNA processing machinery. With the role of cellular miRNAs becoming evident in various aspects of the viral life cycle, ranging from replication [82] to latency [83], the need to focus on stages that are critical to viral activation is important. Based on a recent study wherein HIV-1 was shown to suppress the expression of an miRNA linked to the regulation of P/CAF [61], we proceeded to determine whether the HTLV-1 Tax is able to interfere with the host cellular miRNA machinery and to verify whether any of the downstream targets of the miRNAs affected by Tax modulation are involved in chromatin remodeling. Because each cell type has its own repertoire of miRNAs [70], the newly constructed CD4^+^ T-cell line with a stably integrated HTLV-1 LTR provided the ideal model in which to examine the hypothesis.

The miRNA microarray results and the subsequent analysis using the Web-based tool MicroInspector [62] clearly demonstrated that, following the introduction of Tax into the cell with an integrated viral promoter, there is downregulation of miRNAs that are known to target mRNA members of the HAT family. Out of nine miRNAs specific for P/CAF, three were significantly downregulated (has-miRs-135b, 149, and 872). Similarly, out of the 26 miRNAs specific for p300, three were substantially downregulated (hsa-miRs-149, 872, and 873). In addition, a myeloid-specific miRNA (hsa-miR-223), the expression of which has been shown to be under the control of the transcription factor C/EBP [84], known to be involved in granulopoiesis, was shown to be downregulated and to be highly repressed in the transcription factor array (Table 1). Moreover, the reduced level of miR-223 is associated with an increase in the production, differentiation, and activation of granulocytes that can lead to tissue inflammation as well as leukemia [85]. This observation is particularly exciting because it highlights the involvement of the HTLV-1 transactivating protein Tax in the eventual modulation of the host cellular miRNA pathway. Although the exact mechanism by which Tax causes the observed downregulation of the miRNAs is not known, it is possible that Tax could be acting through the suppression of the type III RNases, Dicer (in cytoplasm) and/or Drosha (in nucleus), that are involved in the biogenesis and processing of miRNAs. The suppression of both of these enzyme complexes could also account for the observed global downregulated pattern following transfection with Tax. With regard to HIV-1, the artificial repression of Dicer and Drosha was shown not only to reduce the processing and maturation of miRNAs but also to enhance HIV-1 replication [61]. In another study, the use of RNA silencing suppressors to mask the cellular RNAi in human cells resulted in increased HIV-1 replication [86]. The transactivating proteins Tat and Tas of HIV-1 and primate foamy virus type 1 (PFV-1), respectively, have been shown to suppress the cellular miRNA-mediated silencing [71], [87]. These studies showcase the participation of the miRNA pathway and its enzymes in repressing HIV-1 and also the ability of the virus to subvert this regulatory pathway to its own advantage. Although no such mechanism has yet been developed for HTLV-1, we demonstrate herein the ability of the HTLV-1 transactivating protein Tax to modulate the cellular miRNA machinery within the CD4^+^ T cell containing a stably integrated HTLV-1 LTR, resulting in a global downregulation of cellular miRNAs, many of which are involved in the regulation of chromatin remodeling HAT factors.

In order to validate our observations, we selected miR-149 and miR-873 that were substantially down regulated and could potentially target p/CAF and p300 based on the software analysis. First, we detected their presence in Jurkat cells and observed significant down regulation in MT-2 cells confirming our miRNA array results in a HTLV-1 infected cell line. To further ensure that these miRNAs could indeed target p/CAF and p300, we over-expressed these in MT-2 cells and observed a sharp decline in the expression of these chromatin-remodeling enzymes. Also, on scoring for virus response, we found a decrease in the levels of viral progeny production in trasfected cells. These observations are first in line to support the virus-miRNA-chromatin concept and require further in depth investigations to tease out the exact molecular mechanisms that lead to viral latency or replication in a given cell type.
