# Expression of Concern: HTLV-1 Tax Mediated Downregulation of miRNAs Associated with Chromatin Remodeling Factors in T Cells with Stably Integrated Viral Promoter

**DOI:** 10.1371/journal.pone.0229498

**Published:** 2020-02-18

**Authors:** The PLOS ONE Editors

After publication of this article [1] several concerns were raised which prompted a reassessment of the reported work. The specific concerns are outlined here, along with additional information provided post-publication by the authors.

- The miRNA data were not deposited at the time of the article’s publication. A dataset was later deposited (GEO GSE39986) as noted in a public Comment; this included a single GPR data file and metadata indicating the experiment had included three biological replicates. Concerns were raised that few data in the GPR file originally deposited with GEO (GSE39986) and/or represented in Fig 4 appeared to indicate signal above background levels, and that one of three triplicate spots for each feature were not included in the public data file. Since, the authors have explained that GSE39986 included first generation miRNAs that are not associated with this article. The GPR data are provided here in Supporting Information S1 and S2 Files.
- The article does not clearly report details as to the number of biological or technical replicates included in the microarray experiment, or whether a dye swap experiment was performed. The authors clarified that two miRNA arrays were used in total; the use of Cy3 versus Cy5 was flipped on the two arrays (i.e. the two represented a dye swap experiment); and the two slides represent independent biological replicates, with RNA extracted from different wells of control and transfected Jurkat cells. Within each slide microRNAs were spotted in triplicate.
- A member of *PLOS ONE*’s Editorial Board consulted in relation to the study noted that the methods by which microarray data were processed and analyzed were not sufficiently reported. The authors noted that TMEV software was used to analyze the data, and this included background corrections to remove false positives, normalization of the data, and filtering to identify results that met criteria of at least 1.5-fold or 1.8-fold expression differences compared to controls. The Academic Editor advised that the information provided, including in the article and in post-publication discussions, are not sufficient to enable others to replicate the data analyses.
- It was raised that the results reported in Fig 4 do not support the statement claiming that miR-873 was identified as potentially targeting p/CAF. The authors do not agree with this concern and stand by their miRNA mimic experiment that confirmed p/CAF as potential target for miR-873 that was also top hit in the MicroInspector analysis.
- Questions were raised as to whether the qPCR experiments using Jurkat and MT-2 cells provide adequate validation of the microarray results, which were obtained by comparing two stably transfected Jurkat lines. The consulted Academic Editor advised that it may be debatable whether the qPCR experiments serve as true validation experiments versus test hypotheses in follow-up to the microarray experiment.
- Questions were raised about GAPDH results reported in Fig 5A. The authors provided quantitative data from these experiments (S3 File). They noted that these experiments were conducted in triplicate, and that the GAPDH Ct data differ between miR-149 and miR-873 experiments because these experiments were conducted at different times and results may have been affected by differences in RNA freeze-thaw cycles.
- The authors noted that the y-axis in Fig 5B was mislabeled and ought to have indicated that mean Jurkat values were set at 1 and MT-2 data were quantified relative to Jurkat values.
- In the right panels of Fig 6A, where authors report western blot experiments to demonstrate miRNA mimic effects on p300 and p/CAF expression, there appear to be vertical discontinuities in background between MT-2 and MT-2+mimic lanes in the p300 and p/CAF blots shown for miR-873. Furthermore, the image quality is poor for both miR-149 and miR-873 panels such that one cannot confirm the integrity of the image data. The corresponding author noted that the original image data to support this figure are no longer available.

The *PLOS ONE* Editors issue this Expression of Concern to alert readers of the remaining questions about the rigor of aspects of the methodology and about compliance with community standards for microarray data availability.

## Supporting information

S1 File Underlying GPR data. (ZIP)

S2 File Full Cluster with scales for both arrays. (JPG)

S3 File Fig 5 raw data and calculations. (XLS)
