# Supplementary figures and images for: Expression of Concern: HTLV-1 Tax Mediated Downregulation of miRNAs Associated with Chromatin Remodeling Factors in T Cells with Stably Integrated Viral Promoter
Source: PLoS One. 2020 Feb 18;15(2):e0229498. doi: 10.1371/journal.pone.0229498 (PMC7028261; doi:10.1371/journal.pone.0229498)

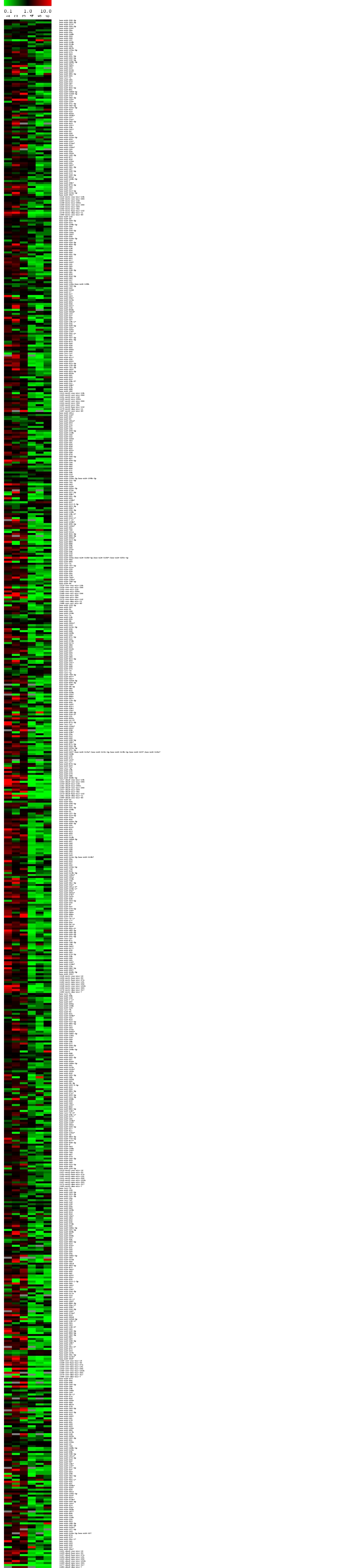

Supplement: S2 File — (JPG) [file pone.0229498.s002.jpg]
